# Efficacy and Safety of Ashwagandha (*Withania somnifera*) Root Extract in Improving Sexual Function in Women: A Pilot Study

**DOI:** 10.1155/2015/284154

**Published:** 2015-10-04

**Authors:** Swati Dongre, Deepak Langade, Sauvik Bhattacharyya

**Affiliations:** ^1^Trupti Hospital and Santati Fertility Center, Cosmos Paradise, Pokhran Road 1, Link Road, Thane, Maharashtra 400 606, India; ^2^Department of Pharmacology, BVDU Dental College & Hospital, Sector 7, C.B.D., Belpada, Navi Mumbai, Maharashtra 400 614, India; ^3^Department of Pharmaceutical Technology, NSHM Knowledge Campus, 124 B.L. Saha Road, Kolkata 700053, India

## Abstract

*Background.* Many women experience sexual dysfunction where there are orgasm disorders and sexual difficulties. Ashwagandha (*Withania somnifera*) is a herb known to improve the body's physical and psychological condition. *Objective.* The purpose of the study was to determine the efficacy and safety of a high-concentration ashwagandha root extract (HCARE) supplementation for improving sexual function in healthy females. *Methods.* In this pilot study, 50 study subjects were randomized to either (i) HCARE-treated group or (ii) placebo- (starch-) treated group. The subjects consumed either HCARE or placebo capsules of 300mg twice daily for 8 weeks. Sexual function was assessed using two psychometric scales, the Female Sexual Function Index (FSFI) Questionnaire and the Female Sexual Distress Scale (FSDS), and by the number of total and successful sexual encounters. *Results.* The analysis indicates that treatment with HCARE leads to significantly higher improvement, relative to placebo, in the FSFI Total score (*p* < 0.001), FSFI domain score for “arousal” (*p* < 0.001), “lubrication” (*p* < 0.001), “orgasm” (*p* = 0.004), and “satisfaction” (*p* < 0.001), and also FSDS score (*p* < 0.001) and the number of successful sexual encounters (*p* < 0.001) at the end of the treatment. *Conclusions.* This study demonstrated that oral administration of HCARE may improve sexual function in healthy women. The present study is registered in the Clinical Trial Registry, Government of India, with a number CTRI/2015/07/006045.

## 1. Introduction

In female sexual dysfunction (FSD), women could have female sexual arousal disorder (FSAD), female orgasmic disorder (FOD), or hypoactive sexual desire disorder (HSDD). Some women may have combined genital and subjective arousal disorder or isolated genital sexual arousal disorder [1]. These disorders result in reduced libido, dryness in vagina, reduced genital perception, reduced arousal, pain during intercourse, and problems to achieve orgasm and are majorly due to neurovascular, hormonal, or psychogenic manifestations [2, 3]. It has been observed that women with sexual problems often turn to alternative therapies and herbal adaptogens as a remedy for diminished sexual desire.

Ashwagandha (*Withania somnifera*) is widely utilized in* Ayurveda*, a traditional system of medicine in India, and is deemed an “adaptogen,” a herb that protects the body from stress and helps the body address the effects of stress. Ashwagandha has been shown to decrease cortisol levels in persons under chronic stress, restore healthy adrenal function, and normalize the sympathetic nervous system [4, 5]. Ashwagandha root extract is used to treat sexual weakness, erectile dysfunction, and performance anxiety in men and has been advocated to ameliorate diminished sexual desire in women and in all forms of sexual dysfunction [6–9], particularly where a depleted nervous system is playing a role.

The contribution of this research is to examine the hypothesis that consumption of a high-concentration ashwagandha root extract (HCARE) may reduce FSD. There are two routes through which this hypothesis may hold. The first route lies in ashwagandha's antistress effect. Chronic stress, experienced often in modern life, may lead to sexual dysfunction in females. A number of literatures have reported the cooccurrence of depression, anxiety, and sexual dysfunction [8–12]. It has been found that under chronic stress women are less motivated and desirous toward sexual activities. Moreover, excessive stress along with anxiety and fatigue leads to sexual arousal difficulties and vaginal pain [10]. A number of studies have confirmed the antistress effect of ashwagandha [4, 13, 14]. Stress is associated with increase in cortisol in the blood, which in turn is associated with gonadal and sexual dysfunction [8]. Ashwagandha reduces serum cortisol level, as has been reported in various clinical studies [4, 13, 14]. The second route to the hypothesis that HCARE consumption may reduce FSD lies in ashwagandha offsetting androgen deficiency syndrome, which is seen as contributing to a lack of sexual desire in some women. Testosterone levels in women tend to decrease with age, and reduced testosterone level may be associated with FSD [15]. Ashwagandha has traditionally been used to treat weakness, erectile dysfunction, performance anxiety in men, and diminished sexual desire in both men and women [6–8]. The herb has been found in men to increase serum testosterone level, decrease follicle-stimulating hormone (FSH) level, and increase luteinizing hormone (LH) production [8, 9]. Ashwagandha may similarly increase testosterone in women and offset androgen deficiency syndrome.

This pilot study is randomized, double blind, and placebo-controlled and aims to assess the efficacy and safety of HCARE in improving FSDS and FSFI in otherwise healthy females with sexual dysfunction and arousal disorder. This is the first study to evaluate the effectiveness of ashwagandha root extract to address FSD.

## 2. Materials and Methods

### 2.1. Study Material

The HCARE used in the present study was KSM-66 ashwagandha, a water-based extract made by Ixoreal Biomed of Los Angeles, California. We chose this extract because our hypotheses center on treatment with HCARE and KSM-66 is currently the highest concentration extract (as assessed by withanolide fraction) available on the market. The use of a publicly available extract like this increases replicability of our procedures by other researchers. Starch powder was used as placebo. We provided the extract and the placebo powder to a local laboratory which then put these into hard gelatin capsules of identical size, shape, color, and texture.

### 2.2. HPLC Conditions

The HPLC analysis of ashwagandha root extract was done by Advanced Analytical Testing Laboratories, North Brunswick, New Jersey. The analysis of withanolides in HCARE was performed on a Waters 515 HPLC system, using a SunFire C18 column of dimensions 250 × 4.6 mm, and 5 *μ*m. The flow rate was 1 mL/min. The solvent system is based on methanol : water (60 : 40). At the end of the run, the column was flushed with 100% methanol for 30 min. The column temperature was 30°C and the injection volume was 20 *μ*L.

Six withanolides were used as marker compounds in this study. 1 mg of a particular marker compound was accurately weighed and transferred into a 10 mL volumetric flask. 10 mL of HPLC grade methanol was poured into this volumetric flask and the solution was sonicated for 15 min or until the compound dissolves completely. From this resultant solution, 1 mL, 2 mL, 3 mL, and 4 mL solutions were transferred into each of four different 10 mL volumetric flasks. The solution was filled up to the mark of each of the flasks with HPLC grade methanol to get a concentration of 10 ppm, 20 ppm, 30 ppm, and 40 ppm, respectively.

50 mg of powdered KSM-66 root extract was transferred to a 50 mL volumetric flask and about 45 mL of methanol was added to it. It was sonicated for 30–45 min with gentle heat in ultrasonic bath. The solution finally was made up with methanol to 50 mL. Prior to injection into the HPLC, this clear solution was filtered using syringe filtration (0.22 *μ*m).

### 2.3. Clinical Study

This pilot study was double blind, placebo-controlled, randomized, and performed in accordance with the ethical guidelines of “Declaration of Helsinki” and approved by the Institutional Ethical Committee of Bharati Vidyapeeth Deemed University, Navi Mumbai 400614, India (date of approval: October 7, 2013). The Ethical Committee notifications followed the Good Clinical Practice (GCP) Guidelines issued by the Central Drugs Standard Control Organization and Ethical Guidelines for Biomedical Research on Human Subjects, issued by Indian Council of Medical Research.

### 2.4. Study Subjects

Subject recruitment was initiated through small clinics in different regions of a large city in India. After obtaining written consent, we recorded demographic characteristics and physicians' assessments from clinical examinations and laboratory diagnostics. Medical personnel performed diagnosis and evaluation for FSD hypoactive sexual desire disorder (HSDD), female sexual arousal disorder (FSAD), female orgasmic disorder (FOD), and combined genital and subjective arousal. The baseline values of systolic and diastolic blood pressure, pulse rate, temperature, respiratory rate, FSFI parameters, and FSDS were assessed. The following inclusion and exclusion criteria were applied.

*Inclusion Criteria.* Inclusion criteria are as follows:

1. Female subjects aged 21–50, in a steady heterosexual relationship for over a year, previously or presently engaged in sexual function for several years.
2. Women who have a male partner with a score of “*not impotent*” or “*minimally impotent*” on the Single-Question, Self-Report of Erectile Dysfunction (Massachusetts Male Aging Study).
3. Women who have a baseline total score of <26 on the FSFI and a baseline total score of >11 on FSDS.
4. Women who have the diagnostic conditions for FSD with one or more of the following disorders:
  - hypoactive sexual desire disorder (HSDD),
  - female sexual arousal disorder (FSAD),
  - female orgasmic disorder (FOD),
  - combined genital and subjective arousal disorder.
5. Women who are willing to engage in sexual intercourse with an intent-to-attain orgasm at least twice/week.
6. Women who provide a written informed consent and can meet all the study requirements.
7. Women who are willing to use condom as a contraceptive measure.
8. Women who are able to speak, read, and write English fluently.

*Exclusion Criteria.* Exclusion criteria are as follows:

1. Women who presented with indication of unsolved sexual distress or exploitation, with FSD caused by untreated endocrine disease, or chronic dyspareunia not associated with vaginal dryness during the previous 12 months.
2. Females with chronic or extensive medical or psychiatric sickness, drug abuse, infertility, and menopause, those who are pregnant or lactating or using hormonal contraceptive pills, and women with known hypersensitivity to ashwagandha.
3. Use of any medications, herbal treatments, or dietary supplements intended to enhance sexual function, in the previous 12 months or during the study.

Those who were finally enrolled into the study were mostly married women, not working in jobs, and coming from affluent households with domestic help. Physicians' notes suggest that the women led lives with high stress from social demands, child rearing, and husbands' high expectations. Qualitative interviews indicated that career women and full-time homemakers were reluctant to participate in this study for an inability to comply with the needs and schedule of the study. All of these factors contributed to the study sample being relatively homogeneous.

### 2.5. Study and Treatment Protocol

The fifty female subjects who met the selection criterion were randomly assigned either to the HCARE-treated group (Group A; *n* = 25) or to the placebo-treated group (Group B; *n* = 25) in a randomized fashion. Subjects in both the groups went through a counselling program consisting of two seminar presentations and an individualized consulting session on addressing FSD. The HCARE treatment was considered as an adjunctive therapy to the counselling sessions. The study duration was 8 weeks for each subject.

### 2.6. Study Drug

HCARE was given in the dose of one capsule of 300 mg twice per day orally after food with a glass of plain water, over a period of 8 weeks. The same protocol was followed for the placebo.

### 2.7. Dose Determination

Based on the withanolide concentration of 5%, we assessed the extract ratio of KSM-66 to be 1 : 10. The traditional dosage of ashwagandha raw root powder is 3000 mg twice a day, as reported in the literature, across a variety of applications. Reducing the traditional dosage commensurately, we arrived at the dosage of 300 mg twice per day.

### 2.8. Concomitant Medication

Any concomitant medication required by the patient was prescribed at the discretion of the investigator and/or the attending clinician in accordance with routine clinical practice at the study site. Only those medicines unlikely to interfere with the study outcomes were allowed to be prescribed.

### 2.9. Efficacy Parameters Evaluated and Measurements

The Female Sexual Function Index (FSFI), the Female Sexual Distress Scale (FSDS), the Sexual Activity Record (SAR), the Patient's Global Assessment of Response to Therapy (PGART), and the Patients Global Assessment of Tolerability to Therapy (PGATT) were used to assess the effectiveness of HCARE to offset FSD. The primary and secondary measures were made at the start and at the 4th and 8th weeks of the study. These primary and secondary outcome ratings were obtained and verified by a competent clinical physician. During the visit of each subject in the 4th week and 8th week, the interviewer reminded her of the responses she gave on the previous measurement occasion, consistent with psychometric methodology to reduce interassay variation.

### 2.10. Primary Efficacy Outcomes

#### 2.10.1. The Female Sexual Function Index (FSFI)

The FSFI is a self-report 19-item survey instrument developed by Rosen et al. [16] and was used to assess FSD extent in each of our subjects. The instrument has domain scores for desire, arousal, lubrication, orgasm, satisfaction, and pain. The FSFI Total Score is a weighted sum of these. The FSFI questionnaire was used at the beginning of the study (to establish the baseline values), at 4 weeks, and at termination of the study period (at 8 weeks).

### 2.11. Secondary Efficacy Outcomes

#### 2.11.1. Female Sexual Distress Scale (FSDS)

The FSDS is a self-report 12-item survey instrument [17] which measures sexually related personal distress in women. The items correspond to various dimensions of sexual distress and are each to be rated according to the assessed occurrence frequency in the previous 30 days.

#### 2.11.2. Sexual Activity Record (SAR)

Assessment of the temporal improvement in sexual activity relative to the baseline values following administration of the specific therapy was based on the Sexual Activity Record (SAR), which measured the encounter frequency of “sexual events” and “successful and satisfactory sexual events.”

### 2.12. Patient's Global Assessment of Response to Therapy (PGART)

PGART was evaluated by the study subjects, at the end of therapy, on a 5-point scale representing the degree of improvement in sexual activity and sexual satisfaction in the following categories: “Excellent response,” “Good response,” “Moderate response,” “Poor response,” and “Worst response.”

### 2.13. Patients Global Assessment of Tolerability to Therapy (PGATT)

Safety was assessed on the basis of adverse events. The subjects were monitored for any adverse drug reactions and illnesses during the study. Any adverse events, either spontaneously reported by the patient or noticed by the physician, were recorded during the trial and forwarded to the primary investigator in a blinded fashion. PGATT was assessed on a 5-point scale with the following scale response categories: “Excellent,” “Good,” “Moderate,” “Poor,” and “Worst.”

### 2.14. Compliance

Subjects were provided “Therapy Kits” containing the medications. At each visit, the investigator/study team noted the number of tablets dispensed and the number of tablets returned by the subject. Any deviations or dose missed was recorded in the* Case Record Form* and* Drug Accountability Log* for enquiry. A patient was considered compliant if ≥80% of medication was consumed according to the prescribed regimen.

### 2.15. Data Evaluation and Statistical Analysis

#### 2.15.1. Analysis Dataset

The recommended practice for clinical trials is that all analysis be done on both the intent-to-treat (ITT) and the per-protocol (PP) datasets. The ITT dataset included all subjects recruited in the study irrespective of their study completion status, whereas the PP dataset included all subjects who completed the study without any protocol violation. In our case, all the subjects completed the study and therefore the ITT dataset and the PP dataset are equivalent.

#### 2.15.2. Data Analysis

Data are reported in terms of mean and standard deviation (SD). Categorical data and discrete data are expressed as numbers with percentages (proportions). Time duration-related changes in each domain score value in the 4th and 8th weeks relative to the baseline value in the ashwagandha group were compared to the corresponding values in the placebo-treated group. Statistical significance was determined using ANOVA tests, both Gaussian and nonparametric Kruskal-Wallis. Bonferroni correction was applied to determine the *p* value thresholds. The treatment variable was a factor in the ANOVA. All testing was done using two-sided tests at alpha = 0.05.

## 3. Results

### 3.1. HPLC Study

**Figure 1:**
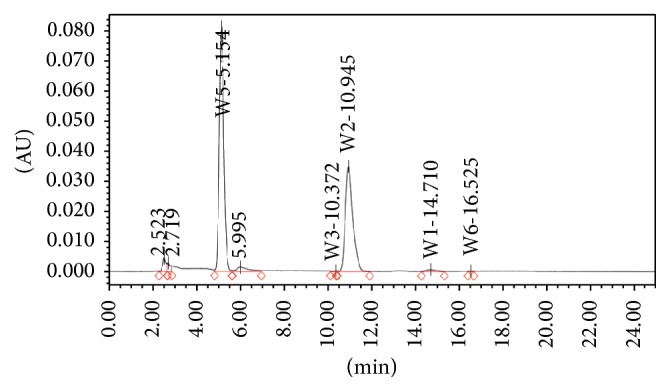
HPLC chromatogram of ashwagandha root extract.

Figure 1 depicts the HPLC chromatogram of HCARE used in this study. The results revealed that the extract contains over 5% withanolides.

### 3.2. Clinical Study

**Figure 2:**
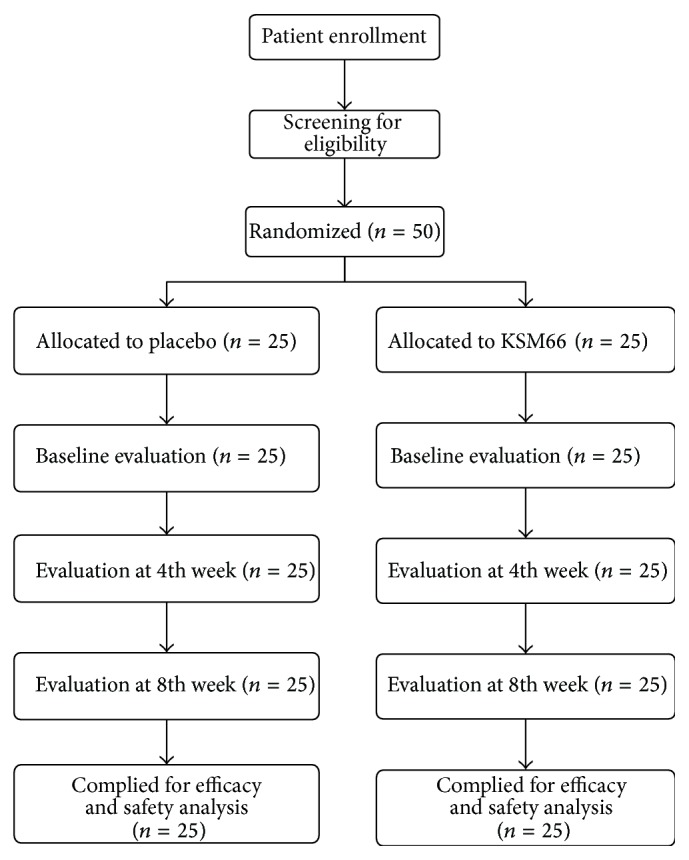
Flow diagram of patient distribution and study design.

The clinical study design and patient distribution are depicted in Figure 2. None of the 50 enrolled women was withdrawn from the study for any reason. The attrition was very low perhaps because the study's administrators, at the time of recruitment, communicated clearly the commitment demanded by the study, thereby discouraging those likely to drop out from enrolling. The general demographic characteristics of the subjects are provided in Table 1; there was no significant across-group difference. The study subjects' mean age was 28.12 ± 5.12 in the HCARE-treated group (Group A; *n* = 25) and 29.44 ± 6.14 in the placebo-treated group (Group B; *n* = 25).

**Figure 3:**
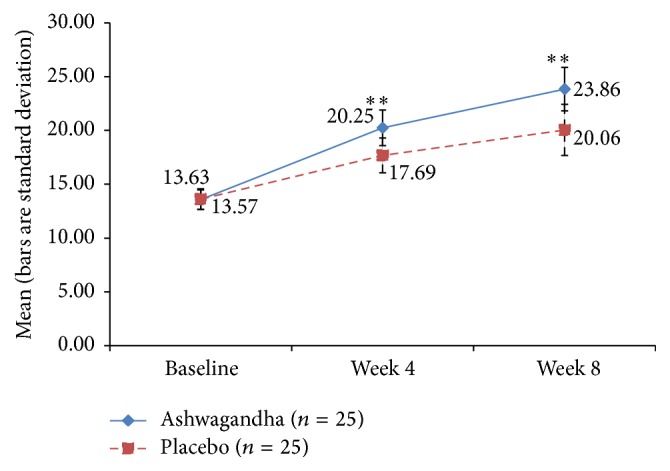
Mean Female Sexual Function Index (FSFI) Total Score in ashwagandha root-treated group and placebo-treated group [^*∗∗*^ *p* < 0.001 ashwagandha root extract treated group versus placebo-treated group].

**Figure 4:**
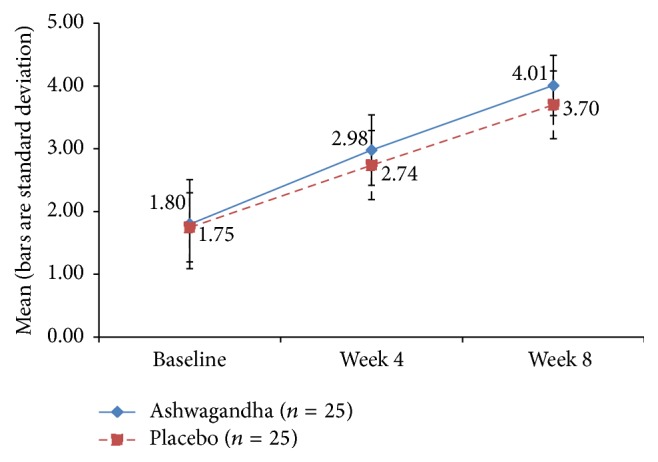
Mean score for “desire” domain of Female Sexual Function Index (FSFI) in ashwagandha root-treated group and placebo-treated group.

**Figure 5:**
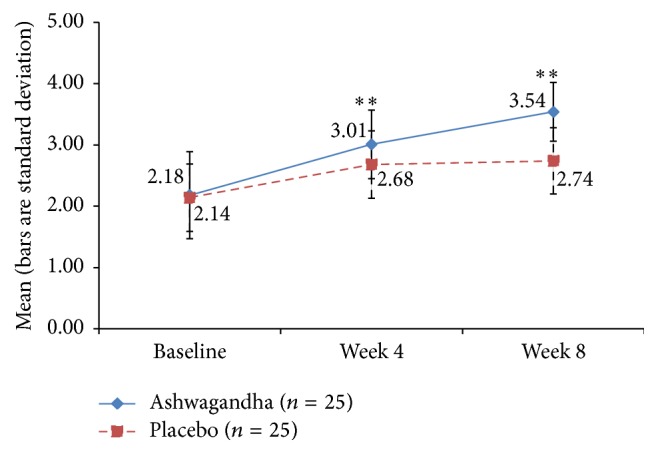
Mean score for “arousal” domain of Female Sexual Function Index (FSFI) in ashwagandha root-treated group and placebo-treated group [^*∗∗*^ *p* < 0.001 ashwagandha root extract treated group versus placebo-treated group].

**Figure 6:**
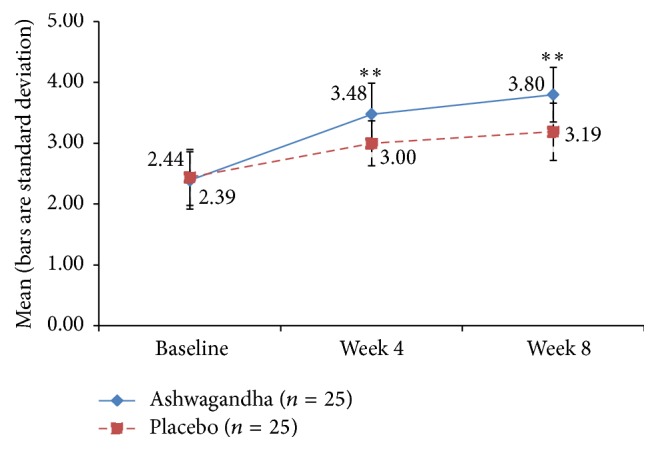
Mean score for “lubrication” domain of Female Sexual Function Index (FSFI) in ashwagandha root-treated group and placebo-treated group [^*∗∗*^ *p* < 0.001 ashwagandha root extract treated group versus placebo-treated group].

**Figure 7:**
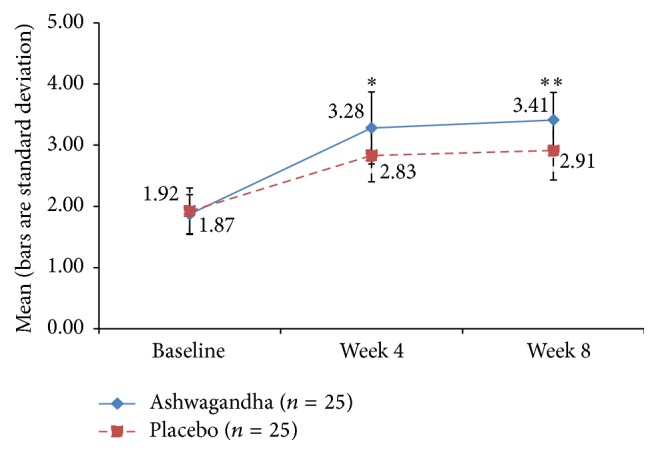
Mean score for “orgasm” domain of Female Sexual Function Index (FSFI) in ashwagandha root-treated group and placebo-treated group [^*∗*^ *p* < 0.01; ^*∗∗*^ *p* < 0.001 ashwagandha root extract treated group versus placebo-treated group].

**Figure 8:**
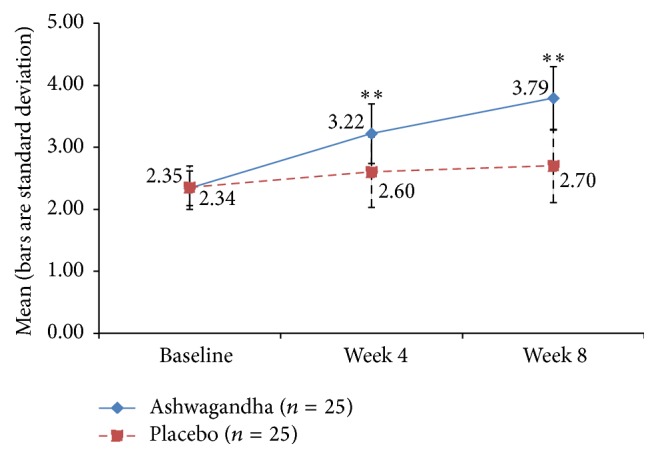
Mean score for “satisfaction” domain of Female Sexual Function Index (FSFI) in ashwagandha root-treated group and placebo-treated group [^*∗∗*^ *p* < 0.001 ashwagandha root extract treated group versus placebo-treated group].

**Figure 9:**
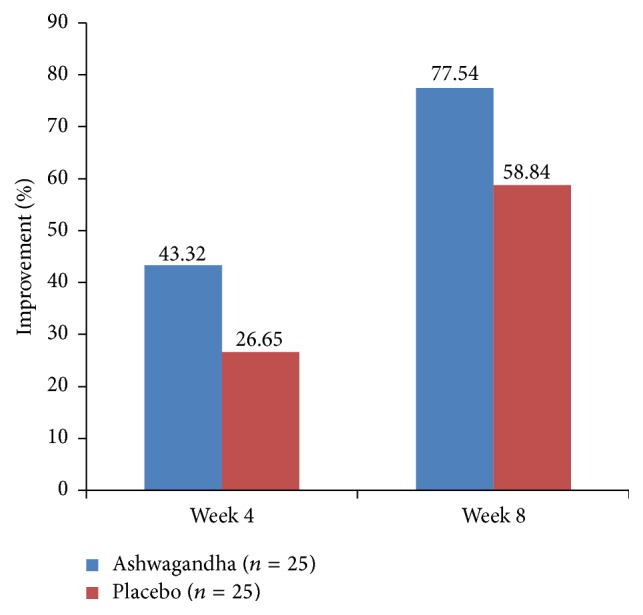
Per cent improvement in the Mean Female Sexual Function Index (FSFI) Score for “pain” domain in ashwagandha root-treated group and placebo-treated group.

The time-specific and group-specific mean values and standard deviations for the key outcome measures are depicted graphically through the points' coordinates and error bars, respectively, in Figures 3–9. The figures visually allow across-time and across-group comparisons of each measure but not an across-group comparison of the overtime increments. Comments on the statistical significance of the latter are made in the text below.

### 3.3. The Female Sexual Function Index (FSFI)

All the women enrolled in the present study had total FSFI scores suggestive of FSD. The FSFI domain scores were suggestive of sexual problems linked to desire, orgasm, lubrication, satisfaction, arousal, and pain. The mean total FSFI score at week 4 of the study period was found to be 20.25 ± 1.66 in Group A and 17.69 ± 1.62 in Group B. At week 8 of the study period, the mean total FSFI score was 23.86 ± 2.02 in Group A and 20.06 ± 2.38 in Group B (Figure 3). The increase in the FSFI Total Score was significantly higher in the HCARE group than in the placebo group at both the 4-week point and the 8-week point (*p* < 0.001 for both comparisons), evidencing that the HCARE led to substantial improvement in FSD in otherwise healthy women.

### 3.4. Desire Domain of FSFI

Figure 4 shows the mean ± SD score for the desire domain of FSFI. The values are comparable, within statistical error, between Group A and Group B at week 4 and week 8, and relative to their respective baseline values. There was no significant difference between HCARE and placebo in the desire domain score increase from baseline to week 4 (*p* = 0.295) and week 8 (*p* = 0.119).

### 3.5. Arousal and Lubrication Domains of FSFI

The mean FSFI arousal domain scores' increments, relative to baseline, were significantly higher in Group A than in Group B at week 4 (*p* = 0.005) and week 8 (*p* < 0.001). For the lubrication domain score, the effects were also strong, with the HCARE group having greater improvement, relative to the placebo, at week 4 (*p* = 0.004) and at week 8 (*p* < 0.001). See Figures 5 and 6.

### 3.6. Orgasm and Satisfaction Domains of FSFI

For the orgasm domain score, the improvement was significantly higher in the HCARE group than placebo, with a significance level of *p* = 0.012 at the 4-week point and *p* = 0.001 at the 8-week point (Figure 7). There was a similar effect for the FSFI domain score for satisfaction (with *p* = 0.001 at 4 weeks and *p* < 0.001 at 8 weeks), not surprising because sexual satisfaction is seen in prior research to be associated with orgasm (Figure 8).

### 3.7. Pain Domain of FSFI

The 4-week and 8-week increments in the mean FSFI domain score for pain were higher under HCARE than placebo (*p* = 0.011 and *p* = 0.002, resp.). However, the difference is not statistically significant after Bonferroni correction (Figure 9).

### 3.8. Female Sexual Distress Scale (FSDS)

**Figure 10:**
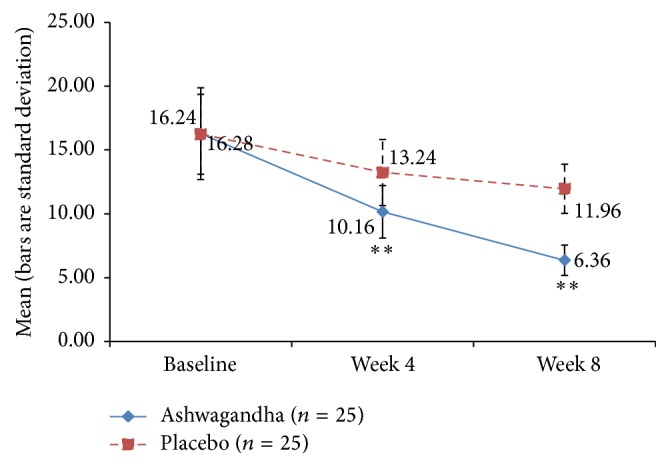
Mean Female Sexual Distress Scale (FSDS) Score in ashwagandha root extract treated group and placebo-treated group [^*∗∗*^ *p* < 0.001 ashwagandha root extract treated group versus placebo-treated group].

The mean FSDS score increased statistically significantly more in the HCARE-treated group (Group A) than in the placebo group, both at week 4 (*p* < 0.001) and at week 8 (*p* < 0.001). See Figure 10.

### 3.9. Sexual Activity Record (SAR)

No significant improvement was observed for the ashwagandha-treated group or the placebo group in terms of the total number of sexual encounters after 4 weeks and 8 weeks. However, significant improvement was observed in the number of successful sexual encounters. The improvement was significantly greater in the HCARE group than in the placebo group but only after 8 weeks (*p* < 0.001) and not after 4 weeks (*p* = 0.056) (Tables 2 and 3).

### 3.10. Patient's Global Assessment of Response to Therapy (PGART)

At the end of therapy (week 8), the study subjects assessed PGART on a 5-point scale capturing the extent of improvement in sexual activity and sexual satisfaction. Of the 25 subjects in Group A, 15 scored the response to the therapy as “Excellent,” 9 as “Good,” and 1 as a “Moderate.” The compliance was excellent for all the patients in both the groups.

### 3.11. Patients Global Assessment of Tolerability to Therapy (PGATT)

No adverse effects of therapy were observed in the HCARE group. All the subjects (*n* = 25) showed excellent tolerability to the therapy. Examination of these data reveals that the HCARE effectively ameliorated some inadequacies in libido and in the psychophysiological variations that characterize FSD.

## 4. Discussion

Sexual expression is a normal and healthy part of human behaviour. Positive sexual experiences are related to health and well-being throughout life; hence, there is a need to think about sexual health as not simply the absence of sexual disorders, but as a key factor affecting the quality of life [18]. FSD is characterized by problems in the psychophysiological variations combined with the “sexual response cycle.” These variations are often due to underlying neurovascular, hormonal, or psychogenic aetiologies [2].

Ashwagandha is shown in the literature to be an anxiolytic, antidepressant, and antistress adaptogen. It has been found effective in stress-induced sexual dysfunctions in animal models. The withanolides, steroidal lactones, are said to be the important phytochemicals of ashwagandha and among the active constituents responsible for the therapeutic efficacies of the plant [19].

There are four findings from the data analysis that are worth highlighting at the outset, the first two relating to the population characteristics and the second two relating to the intertemporal variation in the key outcome measures. First, the FSFI scores we saw were markedly lower than in other reports [20–22]. We attribute these lower scores to restrained attitudes towards sex and towards talking about sex in this specific object pool, owing to its Asian Indian cultural norms. Second, the standard deviations in some of the key outcome measures are lower than in other studies. We attribute this to our subject group being relatively homogeneous and with very similar sociodemographic characteristics, as we pointed out earlier in this paper in Study Subjects. Third, there is significant across-time reduction in FSD (as measured by the FSFI and the FSDS) not only in the HCARE treatment group but also in the placebo group. Recall that both groups' subjects went through a counselling program. This indicates that the counselling program even in the absence of HCARE supplementation helps in FSD. Fourth, HCARE appears useful as an adjuvant to the counselling program in improving many but not all aspects of FSD. We find that the improvement in the FSFI scores from baseline to week 8 is statistically significantly greater under HCARE with counselling than under placebo with counselling, for the number of successful encounters and the FSFI domain scores for orgasm, satisfaction, lubrication, and arousal but not for desire and pain and the number of total sexual encounters.

The explanation for why HCARE treatment was useful in reducing FSD may lie in two pathways: (1) ashwagandha's role in reducing stress, which in turn is associated with FSD, and (2) ashwagandha's role in increasing testosterone which is a factor in androgen deficiency syndrome which in turn is also associated with FSD.

A noteworthy observation in the present study is the substantial placebo-effect evident in the functional measures of various domains of FSFI, in the FSDS scores and in the Event Log Records of successful sexual encounters. This observation confirms previous findings that indicate a marked placebo effect on sexual function of women with sexual difficulties [23, 24]. Several studies have noted major placebo effects. Also noteworthy is the fact that the effects shown under HCARE treatment are strongly significantly higher than in the placebo group. We have been careful and conservative in our statistical significance testing, using recommended tests based on ANOVA under Gaussian and nonparametric assumptions, and applying Bonferroni corrections, thereby reducing the chances of a type I error.

It is important to make some cautionary notes on our study's results so that the reader is conservative and guarded about the conclusion that HCARE supplementation can offset FSD. The *p* values we obtained are considerably lower (and the statistical significances correspondingly higher) than in similar studies. We believe that this is because of three reasons: First, the subject group in our study is more homogeneous than in previous studies, in aspects identified near the end of Study Subjects earlier in this paper. Second, because in weeks 4 and 8 each subject was reminded of and anchored on her responses on the previous measurement occasion, the interassay variation was low in the survey instruments. Third, our sample size of 25 per group was higher than the minimal sample size of 17 calculated (on the basis of an independent pretest sample of size 4) to achieve discrimination power. These three factors likely resulted in substantially lower standard errors, for the key outcome measures, than in previous studies, which led to higher values of the test statistics under the null hypothesis and therefore led to smaller *p* values and higher statistical significance of the effect sizes.

As another cautionary note, we should emphasize that our results should not be interpreted as implying that ashwagandha is an aphrodisiac. In our study, HCARE supplementation failed to improve sexual “desire,” as seen in the nonsignificant improvement in the FSFI “desire” domain score and in the number of total sexual encounters.

## 5. Limitations

A major limitation of the present study design is the relatively homogeneous subject group coming from a specific cross section of society. While a homogenous subject group is good in that it affords greater statistical discrimination power and lower *p* values for any specific effect size, there is the concern that the findings may not generalize to broader cross sections of the population. Future research needs to address this concern by considering subjects with a wider range of demographics, occupations, and socioeconomics. This was a pilot study with only 50 subjects and should be replicated with a larger sample size. Another major limitation is that the study duration is only 8 weeks with three measurement points four weeks apart. A longer duration study with more measurement points may give insight into the temporal trajectory of the effects.

## 6. Conclusion

The results suggest that ashwagandha root extract could be useful for the treatment of FSD. The lack of adverse effects suggests that the extract is safe to consume.

## Figures and Tables

**Table 1:** General demographic characters of the study subjects.

Parameters	Ashwagandha root extract treated group (*n* = 25)		Placebo-treated group (*n* = 25)	
	Mean	Standard deviation	Mean	Standard deviation
Age (years)	28.12	5.12	29.44	6.14
Systolic blood pressure (mm Hg)	122.40	10.12	116.80	14.06
Diastolic blood pressure (mm Hg)	81.60	4.73	76.80	8.02
Pulse rate (per min)	72.24	2.11	70.72	2.23
Temperature (°F)	98.18	0.10	98.20	0.16
Respiratory rate (per min)	17.12	1.54	17.20	1.53

**Table 2:** Mean number of total sexual encounters in ashwagandha root extract treated and placebo-treated groups.

Duration of the study	Ashwagandha root extract treated group (*n* = 25)		Placebo-treated group (*n* = 25)		Unpaired *t*-test
	Mean	Standard deviation	Mean	Standard deviation	“*p*”
Baseline	4.48	1.61	4.64	2.36	0.781
Week 4	5.12	1.01	4.72	1.46	0.266
Week 8	5.12	1.39	4.24	0.72	0.008

Change from baseline	Mean change	% change	Mean change	% change	

Week 4	0.64	1.15	0.08	1.94	0.221
Week 8	0.64	1.96	−0.40	2.48	0.107

**Table 3:** Mean number of successful sexual encounters in ashwagandha root extract treated and placebo-treated groups.

Duration of the study	Ashwagandha root extract treated group (*n* = 25)		Placebo-treated group (*n* = 25)		Unpaired *t*-test
	Mean	Standard deviation	Mean	Standard deviation	“*p*”
Baseline	1.84	0.80	1.96	1.24	0.687
Week 4	3.60	0.96	3.12	1.42	0.169
Week 8	4.16	0.69	3.16	0.75	<0.001

Change from baseline	Mean change	% change	Mean change	% change	

Week 4	1.76	95.65	1.16	59.18	0.056
Week 8	2.32	126.09	1.20	61.22	<0.001
